# Impacts of Heterogeneous Chemistry on Vertical Profiles of Martian Ozone

**DOI:** 10.1029/2022JE007346

**Published:** 2022-11-10

**Authors:** M. A. J. Brown, M. R. Patel, S. R. Lewis, J. A. Holmes, G. J. Sellers, P. M. Streeter, A. Bennaceur, G. Liuzzi, G. L. Villanueva, A. C. Vandaele

**Affiliations:** ^1^ The Open University Milton Keynes UK; ^2^ Space Science and Technology Department Science and Technology Facilities Council Rutherford Appleton Laboratory Oxfordshire UK; ^3^ Planetary Systems Laboratory NASA Goddard Space Flight Center Greenbelt MD USA; ^4^ Department of Physics American University Washington DC USA; ^5^ Royal Belgian Institute for Space Aeronomy (BIRA‐IASB) Brussels Belgium

**Keywords:** Mars, atmosphere, ozone, heterogeneous chemistry, photochemistry, water ice

## Abstract

We show a positive vertical correlation between ozone and water ice using a vertical cross‐correlation analysis with observations from the ExoMars Trace Gas Orbiter's Nadir and Occultation for Mars Discovery instrument. This is particularly apparent during *L*_S_ = 0°–180°, Mars Year 35 at high southern latitudes, when the water vapor abundance is low. Ozone and water vapor are anti‐correlated on Mars; Clancy et al. (2016, https://doi.org/10.1016/j.icarus.2015.11.016) also discuss the anti‐correlation between ozone and water ice. However, our simulations with gas‐phase‐only chemistry using a 1‐D model show that ozone concentration is not influenced by water ice. Heterogeneous chemistry has been proposed as a mechanism to explain the underprediction of ozone in global climate models (GCMs) through the removal of HO_*x*_. We find improving the heterogeneous chemical scheme by creating a separate tracer for the HO_*x*_ adsorbed state, causes ozone abundance to increase when water ice is present (30–50 km), better matching observed trends. When water vapor abundance is high, there is no consistent vertical correlation between observed ozone and water ice and, in simulated scenarios, the heterogeneous chemistry has a minor influence on ozone. HO_*x*_, which are by‐products of water vapor, dominate ozone abundance, masking the effects of heterogeneous chemistry on ozone, and making adsorption of HO_*x*_ have a negligible impact on ozone. This is consistent with gas‐phase‐only modeled ozone, showing good agreement with observations when water vapor is abundant. Overall, the inclusion of heterogeneous chemistry improves the ozone vertical structure in regions of low water vapor abundance, which may partially explain GCM ozone deficits.

## Key Points

- Observed ozone and water ice profiles show a positive vertical correlation, contrary to global ozone and water vapor column anti‐correlation
- Heterogeneous chemistry increases ozone abundance at altitudes where water ice is present, matching locations where ozone is underpredicted
- High hydroxyl radical abundance, assumed proportional to high water vapor abundance, masks the effects of heterogeneous chemistry on ozone

## 1 Introduction

### 1.1 Background

Ozone in the martian atmosphere was first detected in 1969 by Mariner 7 and later by Mariner 9. It was found to vary seasonally in both hemispheres, with a greater abundance in local winter which decreased during local summer (Barth et al., 1973). Other instruments, such as SPICAM (Spectroscopy for the Investigation of the Characteristics of the Atmosphere of Mars) aboard Mars Express (Bertaux et al., 2006), MARCI (Mars Color Imager) aboard Mars Reconnaissance Orbiter (Bell et al., 2009), and NOMAD (Nadir and Occultation for Mars Discovery) aboard the ExoMars Trace Gas Orbiter (TGO) (M. R. Patel et al., 2017; Vandaele et al., 2018) later confirmed the seasonal variability, and observations show that the highest abundance of ozone occurs at high latitudes (>45°N/S) (e.g., Clancy et al., 2016; Khayat et al., 2021; M. R. Patel et al., 2021; Perrier et al., 2006).

Martian ozone is a trace gas highly sensitive to direct (photolysis) and indirect (reactions with photolyzed products) photochemical reactions. Due to its sensitivity to other chemical species and its relatively short lifetime (2–3 hr on the dayside), ozone is often a useful indicator of chemical reactions in the atmosphere (Clancy & Nair, 1996). One of the main destructive pathways of ozone is via reactions with hydroxyl radicals (OH, HO_2_, and HO_*x*_) and atomic hydrogen, H, which are by‐products of water vapor photolysis (Clancy & Nair, 1996; Lefèvre et al., 2004; Shimazaki & Shimizu, 1979). HO_*x*_ are highly reactive and cause a set of chain reactions which produce more HO_*x*_ and lead to further ozone destruction. Their high reactivity makes them a key component in understanding the stability of the martian atmosphere as they catalyze the recombination of atomic oxygen and carbon monoxide to form carbon dioxide (Clancy & Nair, 1996; McElroy & Donahue, 1972). The destructive pathway of ozone caused by HO_*x*_ results in a photochemical anti‐correlation between ozone and water vapor. As a result, the seasonal variation in ozone is influenced by the fluctuation in water vapor throughout the year. The seasonal cycle and asymmetric distribution of water vapor between the two hemispheres leads to a larger ozone total column abundance at high southern latitudes during southern winter, compared to high northern latitudes during northern winter (Montmessin & Lefèvre, 2013; Perrier et al., 2006). The highest recorded total column abundances of ozone have occurred at latitudes >55°S between the northern spring equinox and the northern autumnal equinox (*L*_S_ = 0°–180°). Global climate models (GCMs) are also in agreement with these findings (Clancy et al., 2016; Holmes et al., 2018; Lefèvre et al., 2004; Perrier et al., 2006).

Water ice has been observed throughout most of the martian year in the polar regions (e.g., Giuranna et al., 2019; M. D. Smith, 2004; Stcherbinine et al., 2020; Wolff et al., 2019). During northern and southern winters, water vapor condenses over the polar regions and forms water‐ice clouds known as the North and South Polar Hood respectively (Benson et al., 2010, 2011). The North Polar Hood extends to the pole and persists through the martian year, reaching up to 1.0 opacity between *L*_S_ = 0° and 180° (Giuranna et al., 2019). The South Polar Hood is typically thinner and decreases during the southern summer at *L*_S_ = 220°–330°. Between *L*_S_ = 0° and 180° water ice opacity at the South Polar Hood reaches up to 0.4, although, unlike its northern counterpart, does not extend to the southern pole (Giuranna et al., 2019).

Clancy et al. (2016) and Daerden et al. (2019) showed that the greatest underprediction of ozone in Mars GCMs occurs between *L*_S_ = 0° and 180° at high (>60°N/S) latitudes, relative to MARCI observations. Lefèvre et al. (2008) found that ozone was underpredicted throughout the year across all latitudes, although the largest differences between the modeled and observed data from SPICAM were also between *L*_S_ = 0° and 180° at high northern and southern latitudes. M. R. Patel et al. (2021) showed that the greatest underprediction of ozone occurs in the southern hemisphere at low altitudes also between *L*_S_ = 0° and 180°.

Due to the chemical sensitivity of ozone, an ozone deficit can be used as a proxy for missing or undeveloped chemical reactions in atmospheric models (Lefèvre et al., 2008; Nair et al., 1994). Anbar et al. (1993) suggested ozone abundance could be increased by adding heterogeneous chemistry, by incorporating the adsorption of HO_*x*_ onto the surface of water ice. They simulated this using a 1‐dimensional (1‐D) model, finding that the water ice acted as a sink for HO_*x*_, reducing the destruction rate of ozone and resulting in an enhanced ozone abundance. Lefèvre et al. (2004) mentioned that heterogeneous chemistry could increase simulated ozone abundance. This was further developed by Lefèvre et al. (2008) who included 2 heterogeneous reactions with OH and HO_2_ in their GCM and found that the addition of these reactions increased the total ozone column abundance. The results were in better agreement with SPICAM total ozone column measurements than the gas‐only simulation, although the GCM overpredicted low latitude ozone during the aphelion season (between *L*_S_ = 60° and 150°). However, Clancy et al. (2016) found that simulated ozone with heterogeneous chemistry disagreed with MARCI total ozone column and, using the relationship between ozone and water ice as a proxy for heterogeneous reactions, found that the observed and simulated data had different correlations between ozone and water ice. They concluded that the observations did not show sufficient evidence of heterogeneous processes.

Lefèvre et al. (2021) used an adaptive semi‐implicit scheme (ASIS) from Cariolle et al. (2017) to improve their chemical timestep and stability of the GCM. This study used a more realistic water ice optical depth than previous such studies, which tended to overestimate it. These changes improved the agreement between simulated and SPICAM total ozone column at high northern latitudes. They found that the inclusion of heterogeneous chemistry enhanced ozone abundance at high northern latitudes and was in strong agreement with SPICAM observations. The simulated water vapor was also in good agreement SPICAM, although water vapor was overpredicted in some regions (e.g., low latitudes in aphelion, southern latitudes during southern summer). It is a key species for investigating the effect of heterogeneous chemistry since it is directly related to HO_*x*_ abundance. For Lefèvre et al. (2021), ozone abundance was still underpredicted in some low water vapor abundance scenarios, but matched when water vapor abundance was higher (>1 pr‐μm).

The adsorption of HO_*x*_ onto water ice is a physical process rather than chemical, and thus the method for modeling heterogeneous reactions is ambiguous as there are no definitive chemical reactions. Lefèvre et al. (2008) did not define any products for the heterogeneous reactions, while Lefèvre et al. (2021) later included oxygen and water vapor as products to conserve mass. Modeling of ozone has shown mixed results in explaining observed data and there are still many outstanding problems (Clancy et al., 2016; Lefèvre et al., 2008, 2021). All heterogeneous chemistry studies have used ozone total column abundance, which has a major drawback of only revealing the net difference in ozone throughout the column. In contrast, vertical profiles show the full vertical distribution of species (e.g., ozone and water ice) and, since the species can be viewed as mixing ratios rather than abundances, the relationship between ozone and water ice can be studied with equal weighting across all altitudes. Confirming the presence of ozone and water ice at the same altitudes is crucial to investigating the relationship between the two species as, without defining the vertical distribution, it is difficult to verify any impact heterogeneous reactions have on ozone. Determining the chemical impacts of heterogeneous reactions on both the total abundance and vertical distribution of ozone is essential to understanding how ozone is expected to vary indirectly under the influence of heterogeneous reactions.

### 1.2 Outline

This study uses a combination of statistical analysis and 1‐D modeling to quantify the impacts of heterogeneous reactions on ozone vertical profiles under different circumstances. We analyze observed ozone and water ice vertical profiles at high (>45°) northern and southern latitudes between *L*_S_ = 0° and 180° using a vertical cross‐correlation analysis to determine the relationship between observed ozone and water ice. We then use a 1‐D model with an improved heterogeneous chemical scheme to compare vertical profiles of simulated ozone with and without the heterogeneous chemistry to determine the impact of heterogeneous reactions. We compare a high and a low water vapor abundance scenario to replicate the atmospheric state in the northern summer and southern winter respectively, and assess the influence of heterogeneous chemistry on ozone under such conditions.

Section 2 describes the vertical profiles from NOMAD, the vertical cross‐correlation analysis, and the improved chemical scheme used in the 1‐D model. Section 3 describes the results of the cross‐correlation analysis between ozone and water ice, and the ozone variation in the 1‐D model in low and high water vapor abundance scenarios. Finally, Section 4 discusses the impact of water ice on ozone, and the influence of water vapor on heterogeneous chemistry, before summarizing the conclusions and implications of the study.

## 2 Methods

### 2.1 NOMAD Profiles

Ozone and water ice vertical profiles used in this study are derived respectively from the UVIS (UV‐Visible) and SO (Solar Occultation) spectrometers on the NOMAD instrument aboard TGO, described in M. R. Patel et al. (2021) and Liuzzi et al. (2020). Data cover high latitudes (>45°N/S) from *L*_S_ = 0° to 180° MY 35 and, as there is little zonal variation, all longitudes are included together. As no water vapor data is available <30 km between *L*_S_ = 0° and 30°, it is not possible to estimate the water vapor column and determine whether it would be considered a “high” or “low” water vapor category. Therefore, data between this *L*_S_ period are omitted for the high northern latitudes analysis.

Between *L*_S_ = 30° and 180° at high northern latitudes, ozone abundance is much lower (up to 0.1 ppmv) and thus there are fewer ozone profiles which meet the minimum requirement for the analysis (Table 1). This still leaves 300 profile pairs which meet the conditions set for the cross‐correlation.

**Table 1:** Total Number of Vertical Profiles for High Northern and Southern Latitudes, Followed by the Number of Profiles Which Both Have at Least 1 ppmv of Water Ice, 0.03 ppmv of Ozone, and a Minimum of 6 Datapoints in Each Profile

Latitude (°)	Total profiles	Profiles used	Positive correlations	Percentage positive
≥45N	383	249	90	36.1%
≥45S	711	564	314	55.7%

*Note.* Positive correlations are only included if the altitude displacement is within ±10 km.

Solar occultations profile the atmosphere at the terminator and can occur up to 24 times per sol. Ozone is retrieved within the Hartley Band between wavelengths 240 and 320 nm, while water ice is retrieved using five diffraction orders ranging between 2.2 and 4.3 μm. See M. R. Patel et al. (2021) and Liuzzi et al. (2020) for the full retrieval process for ozone and water ice respectively.

### 2.2 Vertical Cross‐Correlation

We conduct a cross‐correlation between ozone and water ice vertical profiles retrieved from the NOMAD instrument to assess the vertical relationship between the two species. In terrestrial studies, this technique is often used between two time series to determine whether there is a correlated time lag between the variables, along with the nature of this time displacement (e.g., Arattano and Marchi, 2005; Peppa et al., 2017). It is often used when one variable is expected to influence the other and there may be a delay in the response. By displacing the altitude, any vertically displaced patterns between the ozone and water ice can be identified, which, due to the suppression of HO_*x*_ in water‐ice clouds, may impact ozone at different altitudes. In addition, water‐ice clouds can span several kilometers and, with a standard Pearson's correlation (Chatfield, 1983), any increase in ozone which does not span the full altitude range of the water‐ice cloud would not be consistently detected. By conducting a cross‐correlation, any variation in ozone within the water‐ice cloud may be detected within a few kilometers displacement. Furthermore, a standard correlation may not detect any relationship if the vertical profiles of water ice and ozone are displaced at differing altitudes due to any interpolation error, while the cross‐correlation is able to capture this with the vertical lag.

Each cross‐correlation conducted on a pair of vertical profiles produces multiple correlations at different altitude displacements. In this study, a 2‐tailed Student's *T*‐test is used to assess the *p*‐value of each correlation at a significance level of 5% and, from all the significant correlations, the one with the lowest *p*‐value is selected as the most significant correlation. The *p*‐value is the probability of a result being at least as extreme as the observed datapoint, with the assumption that the null hypothesis is true. In this analysis, it is the probability of obtaining a correlation at least as extreme as the observed correlation between ozone and water ice, given the assumption that there is no vertical correlation between ozone and water ice.

**Figure 1:**
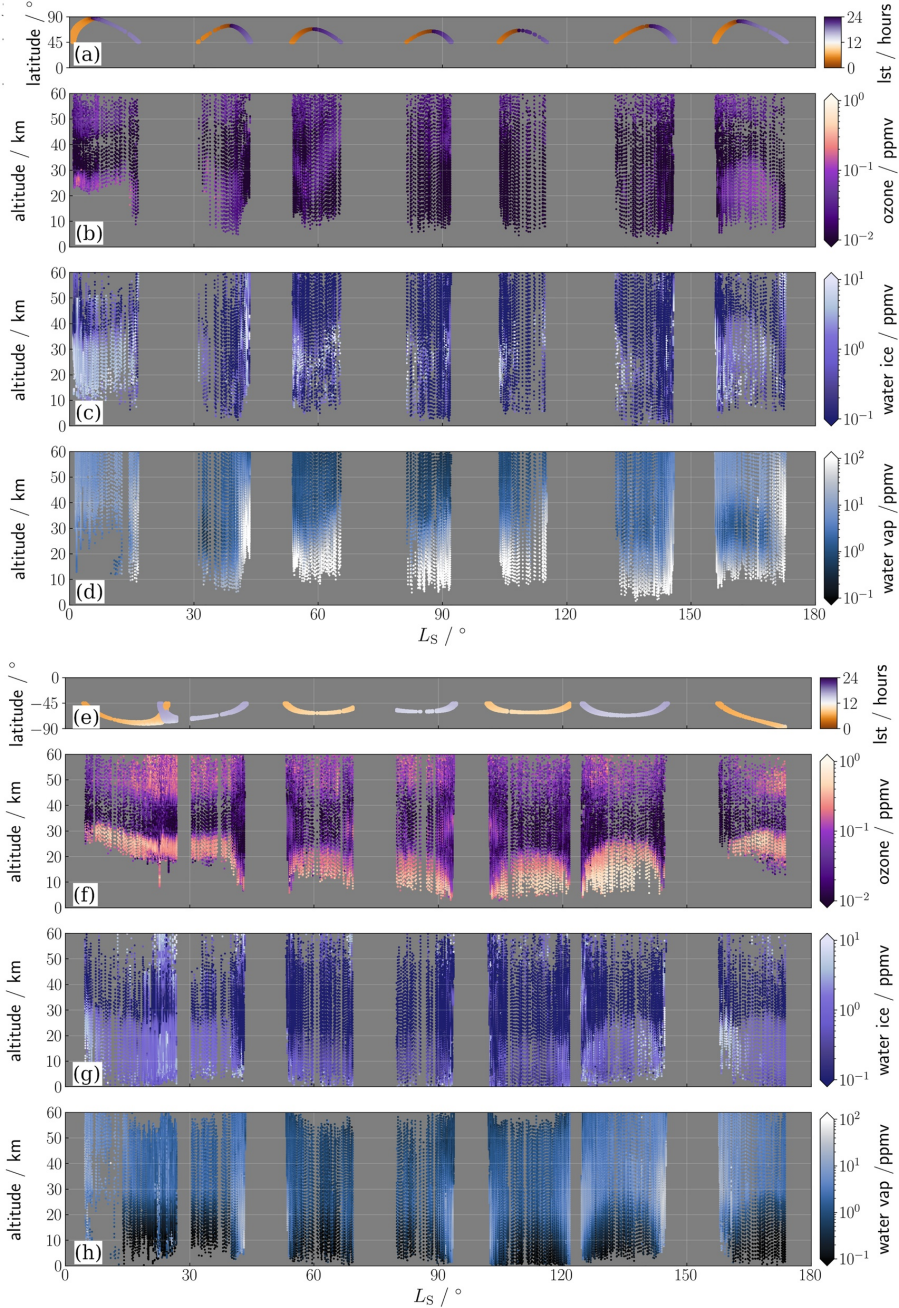
(a) The latitude and local time at which each observed profile is taken. Vertical profiles at high northern latitudes (>45°N) of (b) ozone (M. R. Patel et al., 2021); (c) water ice (Liuzzi et al., 2020); and (d) water vapor (Villanueva et al., 2022) from *L*_S_ = 0° to 180° MY 35 from the Nadir and Occultation for Mars Discovery instrument. (e–h) Equivalent as above but for high southern latitudes (>45°S).

Profiles with no variation and very low volume mixing ratios (vmr) are unsuitable for the vertical correlation analysis as they can produce false correlations that would obscure results in the rest of the analysis. Including such profiles may return false positive correlations between ozone and water ice, for example, two profiles of 0 will produce a perfect correlation. As a result, a minimum condition is set where at least one value in each ozone profile must exceed 0.03 ppmv. Similarly, a minimum threshold of at least one value in each water ice profile exceeding 1 ppmv is used. This restriction enables the vertical variation of ozone in regions of low abundance (such as in the northern hemisphere) to be included in the analysis. Panel b of Figure 1 shows the ozone vertical profiles >45°N for *L*_S_ 0°–180°, MY 35. Panels c and d show the vertical profiles of water ice and water vapor respectively at the same times and locations, while panel a gives the corresponding latitude and local time of the profile. Panels e–h show the same species but for >45°S.

These thresholds are defined graphically, and the sensitivity analysis undertaken indicated that the number of profiles retained for the analysis was robust for the threshold (see Supporting Information S1 for the sensitivity analysis). In addition, a threshold of a minimum of 6 datapoints in each profile is used to filter profiles with poor vertical coverage and which otherwise would be unsuitable for the cross‐correlation analysis.

**Figure 2:**
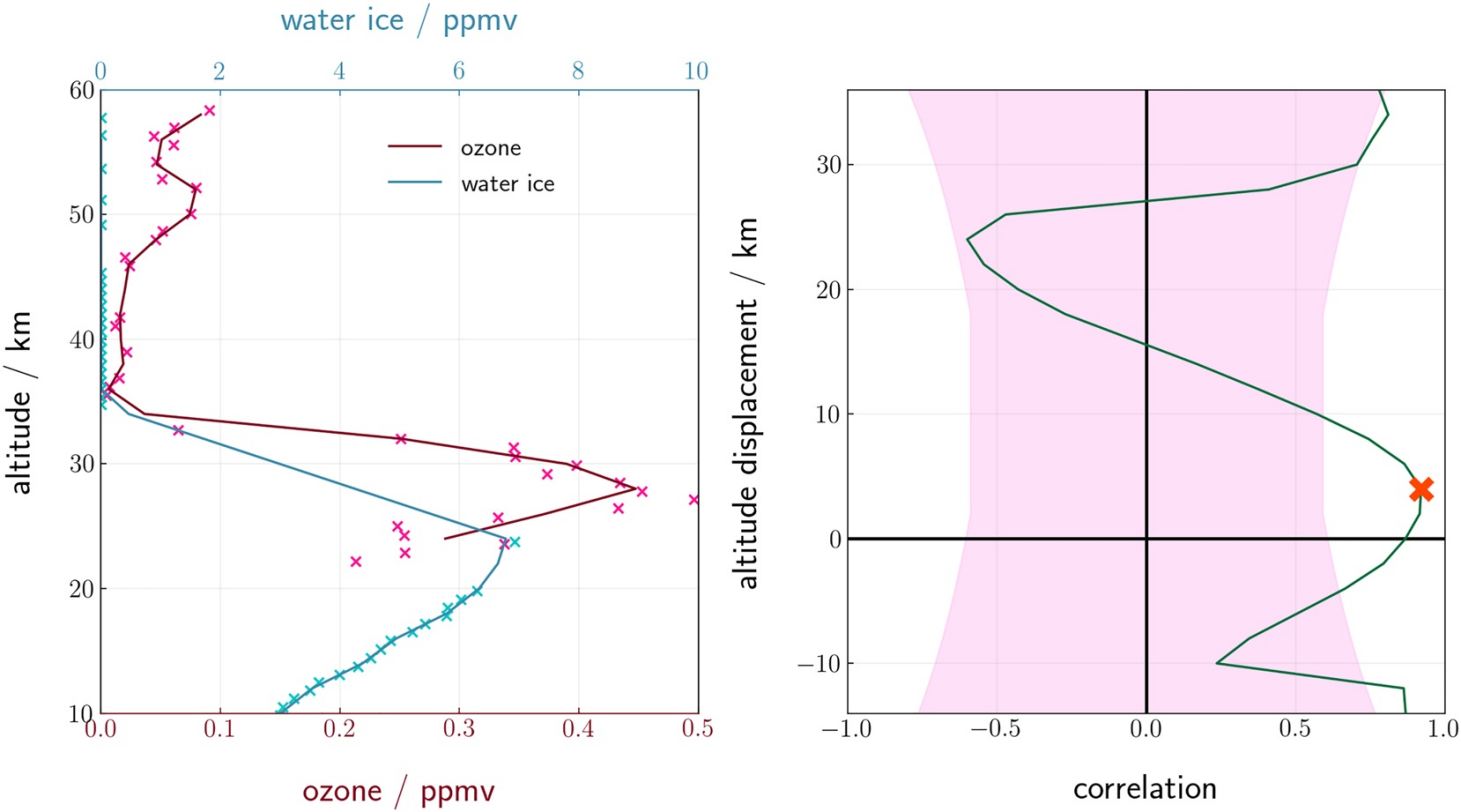
An example of a cross‐correlation analysis between a pair of profiles: (left panel) vertical profiles of (blue) water ice; and (red) ozone, where the crosses are the datapoints and the lines are the interpolated profiles every 2 km: (right panel) the (green) vertical correlation between the profiles across all displacements. A positive displacement of 4 km corresponds to a shifting of the water ice profile up by 4 km. The pink area marks the correlations which are not significant according to a Student's *T*‐test at 5% significance. The red cross indicates the most significant vertical correlation of the cross‐correlation. Profiles are from Nadir and Occultation for Mars Discovery/Trace Gas Orbiter at *L*_S_ = 187°, latitude 70.5°S, 2020.04.21 11:04:57 UTC.

Figure 2 shows an example of the cross‐correlation on a pair of profiles; the maximum correlation between the profiles occurs when ozone is 4 km above water ice (red cross on right panel; correlation 0.85). This corresponds to the increase in ozone between 34 and 28 km, peaking at 0.45 ppmv matching with the increase in water ice between 31 and 25 km which peaks at 7.0 ppmv (left panel). At an altitude displacement <−10 km the decrease in ozone from the peak between 28 and 24 km matches with the decrease in water ice which occurs from 25 to 10 km, causing the correlation at this displacement to increase to 0.8. See the Supporting Information S1 for further details on the methodology used to calculate the cross‐correlation and filter data.

### 2.3 1‐D Model

The 1‐D model used in this work (henceforth referred to as the 1‐dimensional Martian Photochemical Model, 1‐D MPM) is derived from the Open University modeling Group Mars GCM (MGCM), which exists as a collaboration between the Laboratoire de Météorologie Dynamique (LMD), the Open University, the University of Oxford, and Instituto de Astrofísica de Andalućia (Forget et al., 1999).

By using a 1‐D model, the interactions between tracers can be identified purely as chemical interactions without the added complexity of a 3‐D dynamical model, such as the transport of heat, chemical species, and aerosols. The effects of heterogeneous reactions on ozone can therefore be isolated and, by running the model both with and without the heterogeneous reactions, the difference between ozone in both scenarios highlights the direct impact of heterogeneous chemistry on ozone.

The 1‐D MPM is compiled with 70 levels, spaced non‐linearly according to pressure, with 22 tracers and 60 chemical and photochemical reactions. The 1‐D model simulations used to demonstrate the differences between the chemical schemes were spun up for 25 sols to allow water ice to stabilize and produce a steady diurnal variation. Initial starting conditions of temperature, water vapor, water ice, and zonal and meridional winds are taken from the outputs of the MGCM. The MGCM reanalysis data set was assimilated with temperature and dust retrievals (J. A. Holmes et al., 2020) from the Mars Climate Sounder (MCS) instrument aboard the Mars Reconnaissance Orbiter, with photochemistry and the new ASIS chemical scheme.

The full set of chemical reactions and their reaction rates are given in Table 1 of Lefèvre et al. (2004).

The heterogeneous chemical scheme, originally taken from Lefèvre et al. (2021), has been updated to improve the representation of heterogeneous chemistry. The reaction rates used for the heterogeneous uptake of OH and HO_2_ are given in Lefèvre et al. (2008). The products of the improved heterogeneous reactions are treated as separate species rather than recycled into water vapor and oxygen. The improved scheme also includes a third heterogeneous reaction using H_2_O_2_ (Pouvesle et al., 2010). The OH, HO_2_, and H_2_O_2_ are converted into three separate species which are unable to react in any way aside from converting back to their original species. In this heterogeneous scheme, water ice is treated as a sink for HO_*x*_. For OH, this is represented by

(1) $$OH+ice\to ice_{OH},$$

where ice_OH_ is the added species, representing a “sink” for the OH which is adsorbed onto water‐ice particles. The concentration of the ice_HO*x*_ (and ${ice}_{{\text{H}}_{2}O_{2}}$) species only decreases when the tendency of water ice (the rate of change from the previous timestep) is negative and thus water ice sublimates to water vapor. The desorption of the new species is calculated as a proportion of the decrease in water ice due to sublimation relative to the total available abundance of water ice. For example, if 50% of the water ice sublimates to water vapor, then 50% of the ice_HO*x*_ are desorbed to their respective species. The ice_HO*x*_ species have a long enough lifetime to be impacted by vertical transport. To prevent build up, all ice_HO*x*_ species are released if the water ice in the corresponding layer is 0. Initial starting conditions of these new species are set to 0. While previous experiments have been conducted on the dissociation for HO_*x*_ (e.g., W. Smith, 1943), HO_*x*_ dissociation at water ice surfaces has not been studied under martian atmospheric conditions (Cooper & Abbatt, 1996) and, therefore, is not included in this scheme.

Unlike for OH and HO_2_, the adsorption and desorption of H_2_O_2_ onto water ice is a reversible reaction (Pouvesle et al., 2010). The Langmuir adsorption model explains the reversible reaction of the adsorption of species on a monolayer surface. As there is no evidence to suggest the dissociation of ${ice}_{{\text{H˙}2O}_{2}}$, the non‐modified Langmuir equation is used. Therefore, the Langmuir partition coefficient, derived from Pouvesle et al. (2010), is used to calculate the amount of adsorbed H_2_O_2_:

(2) $$\theta_{A}=\frac{N}{N_{\text{max}}}=\frac{K_{\text{Lang}}\;p_{A}}{1+K_{\text{Lang}}\;p_{A}},$$

where *p*_*A*_ is the partial pressure of the species (e.g., H_2_O_2_), *K*_Lang_ is the Langmuir equilibrium constant, and *θ*_*A*_ is the fractional coverage, or the ratio between the gas adsorbed onto the surface (*N*) to the volume of gas adsorbed onto the surface at maximum occupancy (*N*_max_), assuming only monolayer of adsorbate onto the surface (Langmuir, 1918). *K*_Lang_ is calculated by,

(3) $$K_{\text{Lang}}=\frac{K_{\text{linC}}}{N_{\text{max}}}$$

and,

(4) $$K_{\text{linC}}=2.1\times 10^{-5}\exp\left(3800/T\right)$$

where *T* is temperature (K) (Marécal et al., 2010; Pouvesle et al., 2010). Rearranging Equation 2 for *N* and then multiplying by the water ice surface area produces the ${ice}_{{\text{H}}_{2}O_{2}}$ molecules cm^−3^. Pouvesle et al. (2010) conducted the experiment under low temperature and pressure conditions, suitable for extrapolating to the martian atmosphere.

**Figure 3:**
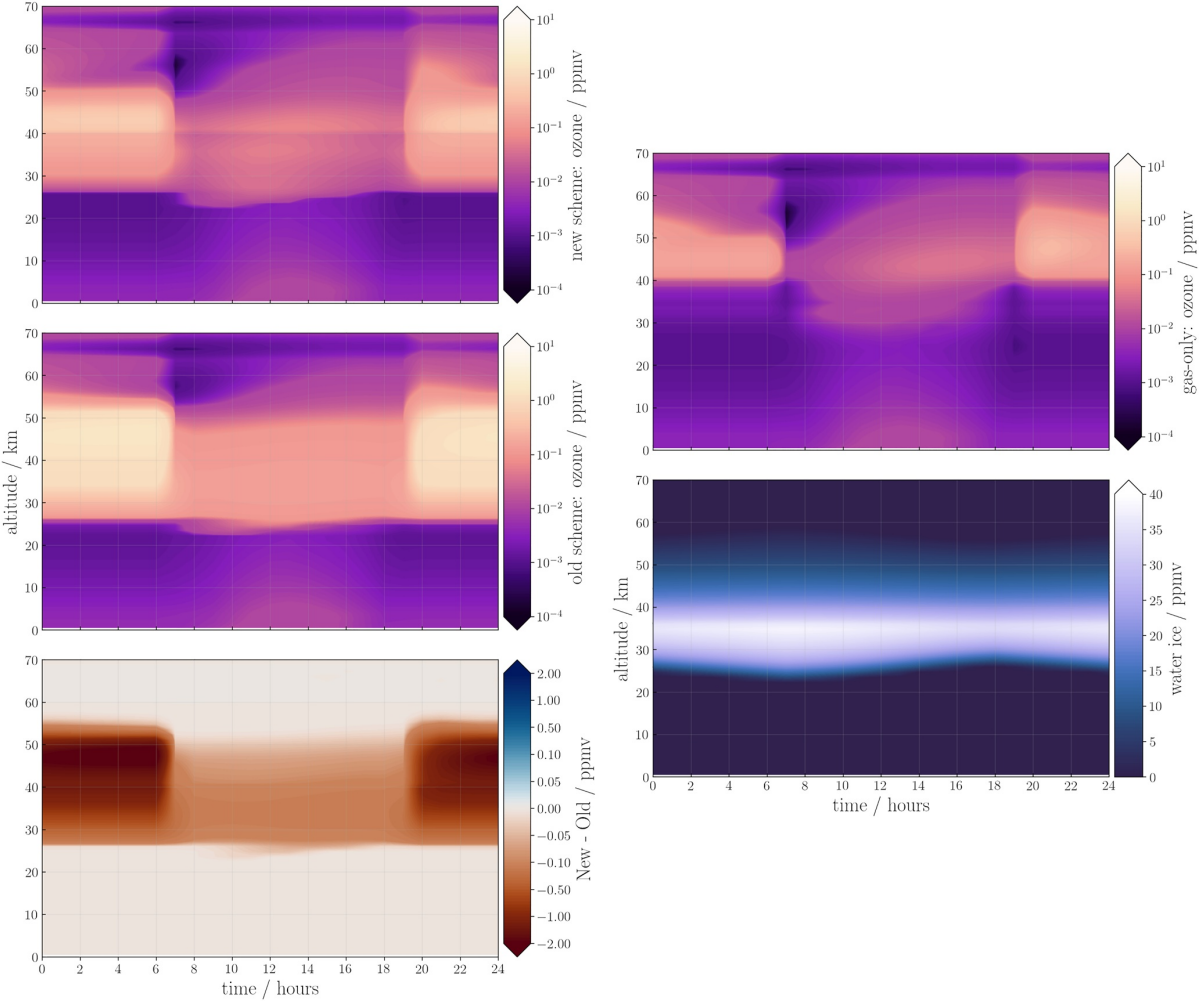
Simulation of ozone abundance over a sol at 0°S, *L*_S_ = 15° with the 1‐dimensional Martian Photochemical Model using (left column); (top) new heterogeneous chemistry scheme; (middle) old heterogeneous chemistry, and (bottom) the difference between the two, and (right column); (top) the gas‐phase‐only ozone; (bottom) water ice.

Figure 3 shows the vertical profile of ozone for the previous and new heterogeneous schemes and the differences between them over one sol at *L*_S_ = 15° (note the non‐linearity on the difference colorbar on the bottom panel). The top right panel of Figure 3 shows the ozone abundance when the model is run with gas‐phase‐only chemistry. The abundance is similar to the new heterogeneous scheme (top left panel), but the ozone layer only exists between 40 and 55 km during the night and extends down to 30 km during the day. This difference is due to the 20–55 km altitude range that water ice forms (bottom right panel), which reduces the ozone destruction rate by the adsorption of HO_*x*_, allowing the ozone abundance to increase.

There is little difference in simulated ozone abundance between the old and new heterogeneous schemes at lower altitudes (<25 km). However, at higher altitudes, the new heterogeneous scheme simulates a lower ozone abundance throughout the sol, between 20 and 55 km (red in the bottom panel). This is likely due to the water ice, which formed at a uniform concentration between 20 and 55 km throughout the sol in both simulations (bottom right panel).

The discrepancy between the ozone abundance in these simulations is likely due to the difference in products from the heterogeneous reactions. The old scheme recycles HO_*x*_ into water vapor and oxygen, both of which can react with other HO_*x*_ radicals and decrease the ozone destruction rate. By contrast, the new scheme holds the HO_*x*_ species in an unreactive state and releases them when ice sublimes.

During the daytime (0600–1800), ozone abundance in the new scheme gradually increases up to 0.1 ppmv, its altitude range spans also increases from 25–45 to 25–60 km (top panel). In contrast, ozone abundance varies little during the day in the old heterogeneous scheme, and has a higher abundance (0.1 ppmv between 0600 and 1800), which ranges between 25 and 50 km. The greatest difference in abundance between the two schemes occurs at nighttime, where the ozone abundance in the new scheme increases to 0.8 ppmv around 50 km, while in the old scheme, ozone increases to >2 ppmv between 25 and 55 km. Overall, the new heterogeneous scheme has a lower ozone total column abundance, due to the differences >30 km.

Two scenarios with high and low water vapor abundance are simulated with the 1‐D MPM to represent high northern and southern latitudes respectively; the scenarios are a simplified representation of high latitude regions and longer temporal periods during the aphelion season. Despite the vertical profiles covering high latitudes, the model scenarios are used only to investigate the ozone variation under different water vapor abundance and not the latitudes themselves. To reduce the parameter differences between the high and low water vapor scenarios, the latitudes and local times are kept consistent at latitude 0° and 1200 local solar time (LST); only the time of year is changed, with the low and high water vapor scenario at *L*_S_ = 60° and *L*_S_ = 180° respectively. The solar insolation is lower in the *L*_S_ = 60° simulation and results in a lower water vapor abundance due to lower temperatures. The vertical structure of temperature remains similar in both scenarios due to the same latitude used, and the local solar time ensures it is a photochemically active time of day. This results in the water‐ice clouds forming at similar altitudes in both scenarios.

Due to limitations in the condensation scheme and the lack of horizontal transport, the 1‐D model is not suitable for simulating tracers in conditions of significant horizontal transport, such as the downwelling of O rich air from the meridional circulation which occurs at this time of year. As a consequence, it is not appropriate to run the 1‐D MPM at high latitudes, and thus only low latitudes are used in this study. The simulation was conducted without microphysics, resulting in the formation of water‐ice clouds whenever the conditions were appropriate (temperature and pressure dependent) and water vapor was available. While this leads to a higher abundance of water ice than if the model were run with microphysics, the simplified scheme is appropriate for the purposes of this study.

For validation, the 1‐D MPM was run at different *L*_S_, local time, and latitudes to investigate the chemical response of ozone from the heterogeneous reactions. The two scenarios used in this study produce similar results to the other simulations and are representative of the ozone variation (not shown). The results from these runs were verified against the MGCM from J. Holmes et al. (2021) and the JPL Caltech 1‐D model from Viúdez‐Moreiras et al. (2019) and found to be in good agreement (see Supporting Information S1 for a full comparison).

The model is run both with and without heterogeneous chemistry, with the gas‐phase only simulation used as a control to investigate the chemical impacts from the heterogeneous reactions.

By definition, the 1‐D MPM is a closed system and does not simulate dynamics or horizontal transport. While this is not necessary for the purposes of testing photochemistry, it means the 1‐D MPM cannot replicate some features which are primarily driven by, for example, thermal tides or downwelling from Hadley cells. The latter of these is particularly important for simulating high latitudes. The temperature in the 1‐D MPM has little diurnal variation, resulting in the water ice abundance staying constant in both abundance and altitude (between 25 and 45 km) throughout a sol, which, due to the mechanism of the adsorbed species, ice_HO*x*_, turning back into their original radicals, means ice_HO*x*_ are never desorbed and turned back into HO_*x*_ species.

In order to resolve this, temperature, surface pressure, and the meridional and zonal wind values are taken from the MGCM and used as inputs into the 1‐D MPM at the beginning of each timestep. The variables are extracted from the MGCM for 2 full sols, with 24 timesteps per sol. After the 1‐D MPM has run through the 2 sols, the extracted temperature, winds, and surface pressures are reset back to the first extracted timestep, and looped through again. This means the 1‐D MPM becomes less accurate over time, as the values taken from the MGCM are for the starting conditions of the 1‐D MPM. However, because these variables, along with the initial water ice and vapor profiles, are extracted from the MGCM, the model only needs 1–2 sols to equilibrate and results are taken from the third sol.

## 3 Results

**Figure 4:**
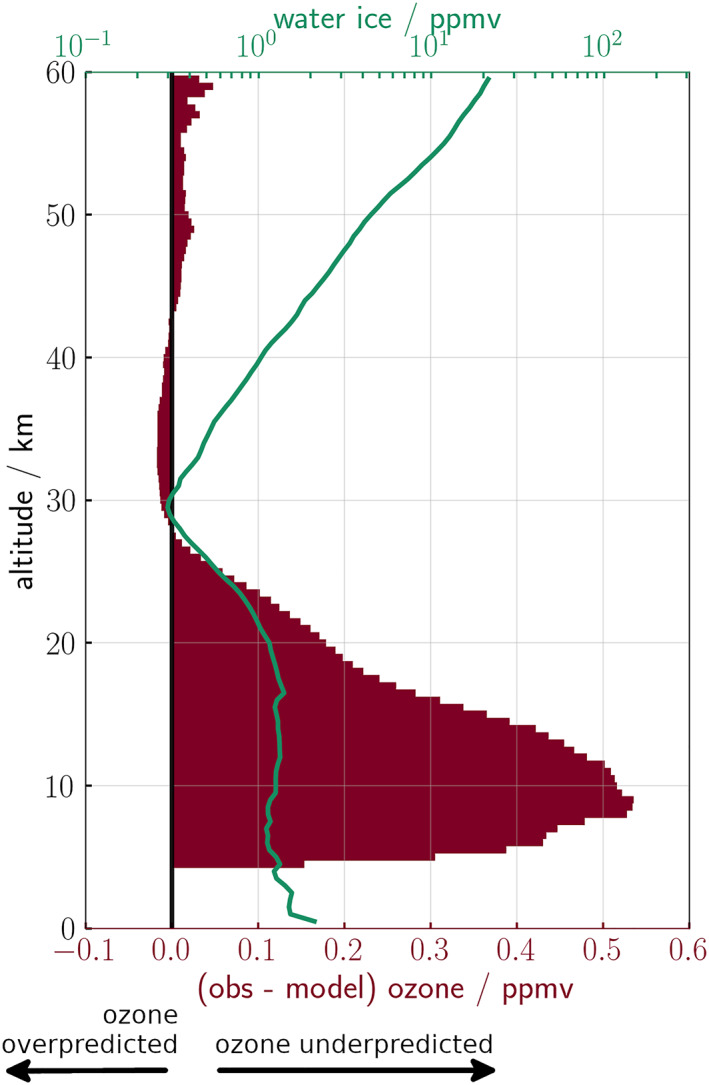
Averaged profiles (212 vertical profiles) >45°S between *L*_S_ = 120° and 150° of (green line) observed water ice, and (red bars) the difference in ozone (observed—modeled). Positive values indicate an underprediction of ozone. Observed profiles are from Nadir and Occultation for Mars Discovery/Trace Gas Orbiter and modeled data are taken from the modeling Group Mars global climate model reanalysis data set.

The two high latitude regions (>45°N/S) of observed vertical profiles from NOMAD used in this study cover *L*_S_ = 0°–180° MY 35 and have contrasting water vapor abundances. The time period incorporates the northern spring equinox to the northern autumnal equinox, where previous studies underpredict ozone for example, Lefèvre et al. (2004), Clancy et al. (2016), Daerden et al. (2019), M. R. Patel et al. (2021), Khayat et al. (2021), and Lefèvre et al. (2021). Figure 4 shows the averaged, observed water ice profiles >45°S between *L*_S_ = 120° and 150° and the ozone difference between modeled and observed profiles from NOMAD/UVIS. Modeled ozone profiles are taken from the MGCM reanalysis data set from J. Holmes et al. (2021) and are simulated without heterogeneous chemistry. An ozone difference greater than 0 ppmv (dark red bars) indicates an underprediction in the MGCM, which follows a similar trend to the water ice abundance. The greatest ozone deficit occurs at lower altitudes (<20 km) and coincides with a higher water ice concentration, while a small deficit occurs above 40 km. Figure 4 is representative of the relationship between the ozone deficit and water ice present throughout the southern hemisphere from *L*_S_ = 0° to 180° (not shown). From the simulations of the 1‐D MPM, ozone abundance increases at altitudes water ice is present. The ozone deficit profile and observed water ice profile in Figure 4 follow a similar structure, which could imply that the model is missing heterogeneous chemistry.

During northern summer, the water ice cap melts, leading to a high abundance of water vapor in the northern hemisphere (Clancy & Nair, 1996; Montmessin & Lefèvre, 2013; Steele et al., 2014). Using NOMAD profiles between 10 and 50 km, the total water vapor abundance is, on average, an order of magnitude larger in the northern hemisphere than the southern (1.37 × 10^−2^ pr‐μm compared to 1.78 × 10^−3^ pr‐μm) (Villanueva et al., 2022). Note that not all occultations extend to the surface, and thus the calculated total column abundance only extends down to 10 km. This is demonstrated in panel d, Figure 1, where between *L*_S_ = 30° and 180° the water vapor reaches concentrations >100 ppmv at 30 km and below. The total column of water vapor is therefore likely much higher, as the lowest altitudes have the greatest contribution to the total column measurement.

### 3.1 Low Vapor Case: Ozone—Water Ice Relationship

**Figure 5:**
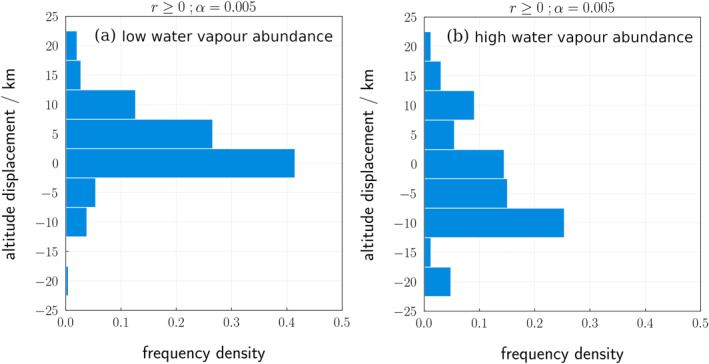
Histogram of altitude displacements for significant, positive, vertical correlations between ozone and water ice observed profiles for (a) southern latitudes (>45°S) *L*_S_ = 0°–180° and (b) northern latitudes (>45°N) between *L*_S_ = 30° and 180°. *α* shows the significance level, and *r* ≥ 0 indicates positive correlations only. Bins cover 5 km, with the *y* axis label denoting the center of the bin (e.g., the 0 km bin includes values from 2.5 to 2.5 km.).

The results from cross‐correlation between the ozone and water ice vertical profiles in the southern latitude region suggest there are significant, positive vertical correlations when the ozone and water ice profiles match at the same altitudes. The relationship between ozone and water ice is more easily discerned when the water vapor abundance is lower. The lower vapor abundance produces a lower HO_*x*_ abundance, which results in a larger relative proportion of HO_*x*_ being adsorbed, thus amplifying the impact of heterogeneous reactions on ozone. The cross‐correlation results demonstrate a clear relationship between ozone and water ice within a vertical displacement range of ±10 km of the two species; panel a of Figure 5 shows a histogram of the altitude displacement from the most significant positive correlations of each profile pair in the southern latitude region. An altitude displacement of 4 km indicates a positive shift in the water ice profile from its original position by 4 km, and thus indicates that the water ice profile matches with an ozone profile which is 4 km above. An altitude displacement of 0 km implies a correlation between the two species at their original position.

In the histogram, there is a sharp peak between −2.5 and 2.5 km with a frequency density of 0.41, implying that water ice and ozone profiles correlate strongly at the same altitude, or when there is a small altitude displacement (<2.5 km). Table 1 shows the number of profiles used in the analysis and the percentage of profiles which have positive correlations within ±10 km at a 95% significance level. If there was no correlation between ozone and water ice, it would be expected that 5% of the profiles would produce a significant, positive correlation due to random chance, as the analysis was conducted at a 95% significance level. Of the profiles suitable for cross‐correlation, 55.7% were positive and within ±10 km altitude displacement, suggesting there is a positive, vertical correlation between ozone and water ice.

The positive correlation between ozone and water ice is continuous throughout the season as can be seen visually in panels f and g in Figure 1. Both species follow a similar latitudinal trend with abundances ranging between 0.2 and 1 ppmv for ozone and 0.5–3 ppmv for water ice between 0 and 30 km. The cross‐correlation matches the variation in ozone and water ice profiles together at 0 km displacement, as both species have a similar vertical variation. In panels f and g of Figure 1, both the water ice and ozone have increased abundances below 30 km, which decrease above this altitude.

During this period, the water vapor abundance is low due to the cold atmospheric temperatures during southern winter. Panel h of Figure 1 shows the vertical profiles of water vapor derived from NOMAD observations from Villanueva et al. (2022), the water vapor ranges from <0.1 to 30 ppmv, with much of the water vapor only observed at altitudes above 30 km.

**Figure 6:**
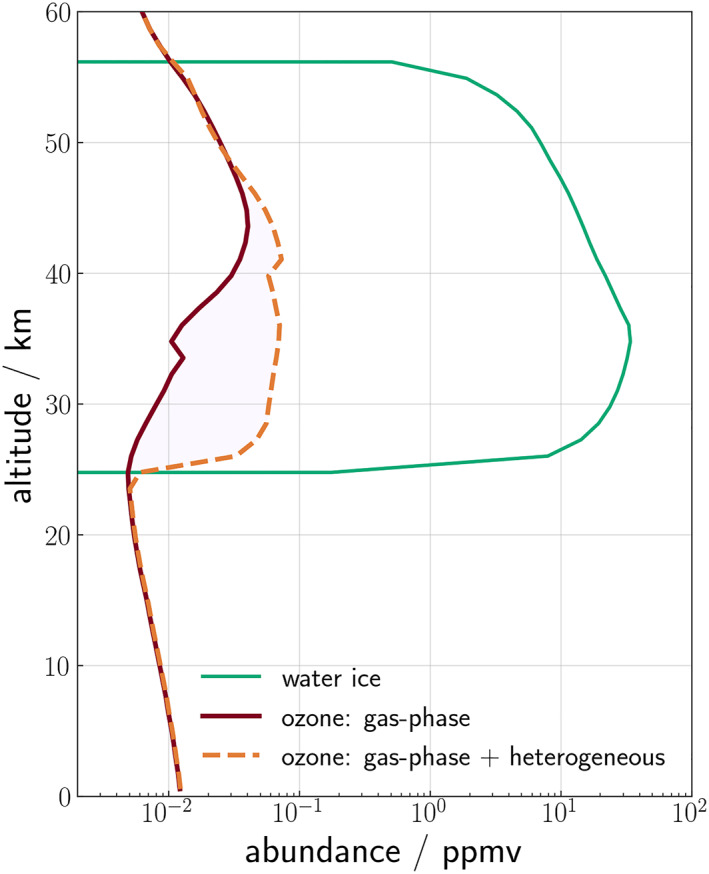
A single modeled vertical profile from the 1‐dimensional Martian Photochemical Model of (dashed orange) ozone from the heterogeneous run, (dark red) ozone from gas‐phase only run and (green) water ice. Profiles are simulated at 1200 local solar time, latitude 0°, *L*_S_ = 15°.

Figure 6 shows a modeled profile from the 1‐D MPM both with and without heterogeneous chemistry taken at 1200 LST, *L*_S_ = 15°, latitude 0° as explained in Section 2. Between 25 and 45 km there is an increase in ozone in the heterogeneous simulation (dashed orange) which is not observed in the gas‐phase‐only simulated ozone (dark red). This increase is due to the heterogeneous reactions which occur when water ice (green) is present. The water ice adsorbs the HO_*x*_ and reduces the HO_*x*_ abundance, which results in a lower ozone destruction rate and enhances the ozone abundance. The peak increase in ozone from heterogeneous reactions in the low water vapor abundance simulations are proportional to 43%–75% of the peak ozone deficit shown in Figure 4. While Figure 6 is not necessarily a realistic profile directly comparable to observations, it demonstrates how the vertical profile of ozone changes in water ice clouds when heterogeneous chemistry is included.

The ozone simulated with heterogeneous chemistry between 45 and 55 km does not exhibit the same response, however, despite the existence of water ice at these altitudes. Indeed, the ozone simulated with heterogeneous chemistry is slightly lower than the gas‐phase‐only ozone. This is due to a decrease in water ice abundance between 45 and 55 km from the previous timestep, which causes the release of previously adsorbed hydroxyl radicals. In the new scheme, water ice uptakes OH and stores it as ice_OH_ if no ice sublimates. This can occur for several timesteps, inbetween which the OH can re‐equilibrate. The sudden release of ice_OH_ leads to a surge in OH, which has been stored from previous timesteps, resulting in more OH than the control (gas‐phase‐only) simulation.

### 3.2 High Vapor Case: No Ozone—Water Ice Correlation

Given that we have found that the altitude displacement between ozone and water ice is likely with ±10 km altitude range in the southern hemisphere, exploring the correlation between ozone and water ice in the northern hemisphere at this altitude range could yield similar results. Panel b of Figure 5 shows the histogram of significant, positive vertical correlations for observed ozone and water ice profiles at high northern latitudes. Unlike in the southern hemisphere, the distribution of altitude displacements in the northern hemisphere is more uniform and there is no clear correlation between ozone and water ice in one altitude displacement range.

In addition to this, there are proportionally fewer significant positive correlations, with only 36.1% of the most significant correlations being positive within ±10 km displacement, compared to 55.7% in the southern latitude region (see Table 1).

As discussed previously, the high northern latitudes between *L*_S_ = 30° and 180° have a higher water vapor abundance than the high southern latitudes (lower panels of Figure 1), as the time period covers the northern summer and the sublimation of the water‐ice cap (Montmessin & Lefèvre, 2013; Steele et al., 2014). To simulate similar conditions of higher water vapor abundance, a 1‐D MPM simulation at latitude 0°, *L*_S_ = 180° is used to investigate the response of ozone to the heterogeneous reactions. The vertical profile from this model run is then compared to another with a much lower water vapor abundance at *L*_S_ = 60°; total column water vapor abundance is 9.3 and 3.8 pr‐μm for the high and low vapor scenarios respectively. Note that these values are the total column abundance for the full vertical column and thus not directly comparable to the abundances calculated for the observed vertical profiles which only include altitudes between 10 and 50 km. The total column water vapor abundances between 10 and 50 km are 2.1 and 0.008 pr‐μm for the high and low water vapor scenario respectively. While these water vapor columns do not match exactly with the observed profiles of water vapor (which extend down to 10 km), the differences in abundance between the two scenarios are of a similar order of magnitude, and the 1‐D MPM simulations demonstrate how the heterogeneous reactions affect ozone under these situations.

**Figure 7:**
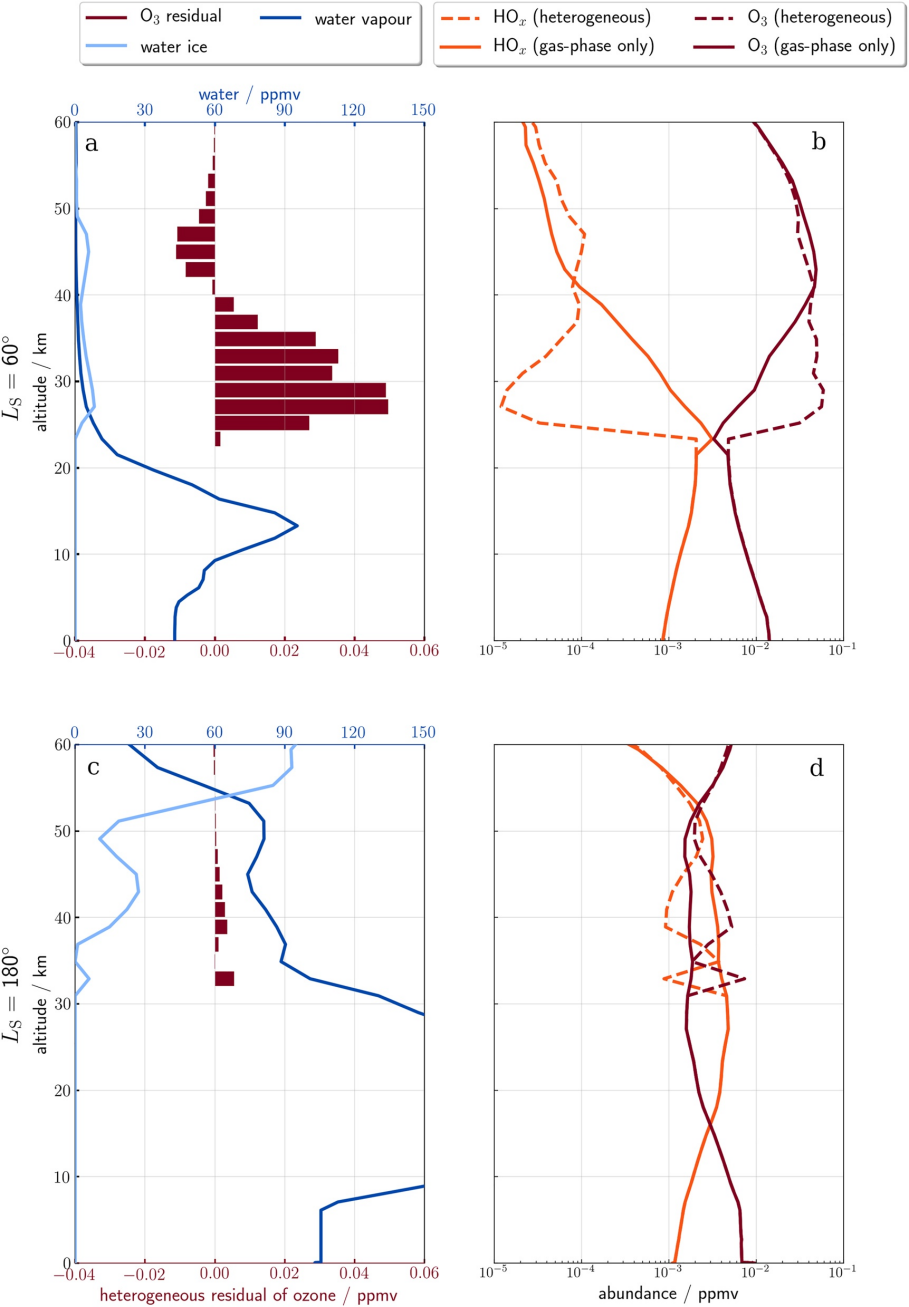
Modeled profiles from the 1‐dimensional Martian Photochemical Modelof (a and b) low water vapor (at *L*_S_ = 60°), and (c and d) high water vapor (at *L*_S_ = 180°) at latitude 0°, local solar time 1200 hr (First column; a and c) vertical profiles of (light blue) water ice, (dark blue) water vapor, and (dark red bars) the ozone residual (calculated by subtracting the heterogeneous ozone from the gas‐phase only ozone). Abundance difference for ozone is on the bottom *x*‐axis and abundance for water ice and vapor is on the top *x*‐axis. (Second column; b and d) vertical profiles of (dark red) ozone and (orange) HO_*x*_ for (dashed) heterogeneous and (solid) gas‐phase‐only simulations. Note the abundances are on a logarithmic scale.

Figure 7 demonstrates the impact of heterogeneous chemistry on ozone abundance for low (*L*_S_ = 60°; panels a and b), and high (*L*_S_ = 180°; panels c and d) water vapor abundances through the change in hydroxyl radicals. Panels a and c show the vertical profile of water ice and water vapor (lines) as well as the ozone residual (bars) which is the heterogeneous simulation minus the gas‐phase simulation. Panels b and d shows the vertical profile of ozone and HO_*x*_ (combined OH + HO_2_). Panel a shows an excess of ozone (dark red bars) from the heterogeneous simulation up to 0.05 ppmv in the low water vapor scenario (shown by the water vapor profile; dark blue) between 25 and 40 km. Panel b shows the ozone (dark red) and HO_*x*_ (OH and HO_2_; orange) abundance for the control (solid) and heterogeneous (dashed) simulation; the large difference between the two HO_*x*_ abundances (over two orders of magnitude difference between 25 and 30 km) is a direct result of the heterogeneous reactions caused by the presence of water ice at these altitudes. In contrast, panel c only has an ozone residual of up to 0.005 ppmv in the high water vapor abundance scenario (e.g., >50°N, Crismani et al., 2021) despite having a larger water ice abundance (light blue) of 30 ppmv compared to 10 ppmv. The HO_*x*_ abundance is over an order of magnitude higher in the control simulation between 30 and 45 km and the adsorption of HO_*x*_ onto water ice is proportionally much smaller than the lower water vapor scenario; between 30 and 35 km, HO_*x*_ decreases from 3 × 10^−3^ to 1 × 10^−3^ ppmv, while in the low vapor scenario HO_*x*_ decreases from 3 × 10^−4^ to 1 × 10^−5^ ppmv despite the water ice abundance being three times less.

## 4 Discussion

### 4.1 Impact of Heterogeneous Chemistry on Ozone

The positive vertical correlation between ozone and water ice in the southern latitude region can be used as a proxy for the presence of heterogeneous reactions. The results of the correlation analysis are supported by the 1‐D MPM simulations which, in Figure 6, demonstrate the difference between ozone profiles in the presence of water ice, both with and without heterogeneous chemistry. The increase in ozone vmr due to heterogeneous reactions occurs at the same altitudes water ice is present in. According to the simulated profile in Figure 6, this is between 30 and 40 km.

The increase in ozone vmr due to heterogeneous reactions occurs at the same altitudes as water ice is present, which, in the simulated profile in Figure 6, is between 30 and 40 km. The heterogeneous reactions act as a sink for HO_*x*_, reducing the abundance of HO_*x*_ as the water‐ice surface area increases. As HO_*x*_ reactions with ozone are one of the main destructive pathways of ozone, the decrease in HO_*x*_ vmr ultimately leads to an increase in ozone vmr within the altitudes that water ice has formed.

The adsorption rate of heterogeneous reactions is dependent on the water‐ice surface area, HO_*x*_ molecular density, and temperature. At higher altitudes, all three factors are typically lower due to the exponential decrease in pressure and general monotonic decrease in temperature. The impact of heterogeneous chemistry, therefore, is reduced at higher altitudes. The decrease in molecular density of HO_*x*_ with increasing altitude results in a smaller difference between the simulations, with and without heterogeneous chemistry; this is shown by the decrease in difference of ozone as altitude increases in Figures 6 and 7. This suggests that heterogeneous chemistry has less influence on the ozone abundance at high (>50 km) altitudes, which is reflected in the ozone deficit between simulated and observed vertical profiles as seen in Figure 4. The largest ozone deficit between MGCM simulated and observed profiles coincides with a higher water ice concentration below 30 km, while only a small deficit occurs above 40 km. The positive vertical correlation between ozone and water ice occurs below 30 km; this relationship matches the response observed in the 1‐D MPM heterogeneous simulation, in which ozone increases at the same altitudes water ice forms. Heterogeneous chemistry could therefore explain some (43–75%) of the ozone deficit, as the addition of the heterogeneous reactions increases ozone at the altitudes water ice is present and has a greater impact on ozone abundance at low altitudes. As the heterogeneous reactions do not fully account for the ozone deficit, it is possible that there are other unknown reactions occurring, or the rates of the heterogeneous reactions themselves may be incomplete.

### 4.2 Water Vapor Influence on Heterogeneous Chemistry

The lack of any positive vertical correlation between ozone and water ice at a consistent altitude range in the northern hemisphere suggests there is no relationship between ozone and water ice and, by proxy, a very weak effect of heterogeneous chemistry on ozone. This conflicts with the clear relationship observed between the two species in the southern hemisphere.

One explanation for this could be that, globally, there is no heterogeneous chemistry occurring and the relationship observed in the southern hemisphere is simply an anti‐correlation between ozone and water vapor portrayed through the water ice distribution which, itself, appears to be an inverse of the water vapor distribution (Figure 1). If this were the case, however, the relationship observed at the northern latitudes should be similar to that of the southern latitudes, and there should be no discrepancy between the hemispheres.

These contrasting results between the high northern and southern latitudes therefore require a different explanation. The 1‐D MPM simulations of a high and low water vapor scenario enable an explicit investigation into the chemical impact of the heterogeneous reactions between HO_*x*_ and ozone. Despite the decrease in HO_*x*_ abundance from the control to the heterogeneous simulation in the high water vapor scenario (lower panels, Figure 7), there is little difference in ozone abundance, indicating that the heterogeneous reactions have relatively little impact on ozone abundance in circumstances of high HO_*x*_ abundance. As HO_*x*_ is a by‐product of water vapor photolysis, a high abundance of water vapor can be assumed proportional to a high HO_*x*_ abundance. In the northern hemisphere during northern summer, the water vapor abundance is higher than observed in the southern hemisphere and, by extension, the HO_*x*_ abundance is also likely to be greater. The relative decrease of HO_*x*_ abundance as a result of heterogeneous chemistry has a negligible impact on the abundance of ozone and is unlikely to be detectable through ozone variation. Therefore, ozone and water ice would not be expected to have a positive relationship in scenarios of high water vapor abundance, as heterogeneous chemistry is offset by the additional availability of HO_*x*_ and the ozone abundance does not significantly increase under such circumstances.

Lefèvre et al. (2021) find good agreement between total column abundance of ozone and water vapor between GCM simulations and SPICAM observations when including the old scheme of heterogeneous chemistry. In particular, the simulations match well with the observations between 60°N and 90°N, when water vapor is between 1 and 50 pr‐μm. While the 1‐D MPM simulations show that a higher water vapor abundance results in a weaker effect of heterogeneous reactions on ozone, there are other factors, such as the difference between temperature profiles at low latitudes and the polar regions, which impact the rates of the heterogeneous reactions. Moreover, the old heterogeneous scheme produces more ozone than the new scheme.

As the 1‐D MPM simulations are conducted at low latitudes, the full impact of the heterogeneous reactions cannot be directly extrapolated to the polar regions; extreme low temperatures in the polar regions affect the reaction rates for a number of reactions. In addition, a small increase in the ozone vertical profile can have a varying effect on the total column abundance, depending on the altitudes at which ozone abundance increases. In the polar regions, water ice can form at low altitudes (Liuzzi et al., 2020), which would have significantly greater impact on the total column ozone abundance, compared to water‐ice clouds which form at higher altitudes.

### 4.3 Conclusions

We find that the influence of heterogeneous reactions on ozone is dependent on the abundance of water vapor, which undergoes seasonal and spatial variation. The relationship between the observed ozone and water ice, which is used as a proxy for heterogeneous chemistry, is also expected to vary temporally and spatially.

In the cross‐correlation analysis at high southern latitudes, there is a positive vertical correlation between ozone and water ice at 0 km displacement. In contrast, in the northern latitudes, when there is a much higher water vapor abundance, there is no clear positive correlation. In the 1‐D MPM simulations, the ozone abundance is much lower in the high water vapor scenario and the ozone residual between the heterogeneous and gas‐phase simulation is closer to 0 ppmv, implying that there is a minimal effect (<0.005 ppmv increase) of heterogeneous chemistry on ozone under high water vapor conditions.

The photolysis of water vapor is not the sole factor in the water cycle affecting the global ozone distribution—water ice also appears to influence ozone abundance indirectly through the adsorption of hydroxyl radicals.

The cross‐correlation conducted on the ozone and water ice vmr profiles shows that these two variables are positively correlated in the vertical in regions of low water vapor abundance. This is contrary to previous studies which found an anti‐correlation between the total abundance of ozone and water ice (Clancy et al., 2016). From investigating the ozone variation between 1‐D MPM outputs with and without heterogeneous chemistry, we show that heterogeneous reactions between HO_*x*_ and water ice are a plausible explanation for the positive vertical correlation between ozone and water ice.

Furthermore, heterogeneous chemistry increases the ozone vmr at altitudes water ice forms, and thus could explain some of the ozone deficit in GCMs when compared to observations, which is in agreement with Lefèvre et al. (2008, 2021). While the addition of heterogeneous chemistry may not fully resolve the ozone deficit seen in GCMs, it increases the ozone abundance at locations which currently underpredict ozone, while having a minimal effect on ozone at higher altitudes and in areas of higher water vapor abundance (<0.005 ppmv), where models already show good agreement with observations.

The role of heterogeneous chemistry in the martian atmosphere could extend to other reactions, such as the uptake of ozone onto the surface of dust particles which has been studied in the terrestrial atmosphere (Michel et al., 2003; Mogili et al., 2006). Such reactions in the martian atmosphere could further impact the vertical distribution of ozone by providing an additional sink, particularly in areas of greater water vapor abundance where the adsorption of ozone onto dust is more effective (Mogili et al., 2006).

Heterogeneous chemistry affects the variation in hydroxyl radicals. Therefore, establishing the nature of heterogeneous reactions is important in understanding the variation in hydroxyl radicals, which themselves are crucial to the stability of the atmosphere for example, McElroy and Donahue (1972) and Clancy and Nair (1996). The presence of water‐ice clouds could indirectly affect the recombination of carbon monoxide and molecular oxygen, which is catalyzed by hydroxyl radicals. Future work could include establishing the full spatial and temporal impact of heterogeneous chemistry on ozone and hydroxyl radicals by implementing the improved chemical scheme into a GCM, as well as investigating how the change in HO_*x*_ in water‐ice clouds could impact other species aside from ozone.

## Supporting information

Supporting Information S1

## Data Availability

NOMAD vertical profiles and 1‐D MPM simulations used for the plots in this study can be found in Brown et al. (2022). The full ozone data set can be found in M. Patel et al. (2021). The water vapor data set from Villanueva et al. (2022) can be found at NASA's ExoMars archive (https://psg.gsfc.nasa.gov/apps/exomars.php). Color maps are from Scientific Color Maps Version 7.0 from Crameri (2021).
