# Supplementary material for: Impacts of Heterogeneous Chemistry on Vertical Profiles of Martian Ozone
Source: J Geophys Res Planets. 2022 Nov 10;127(11):e2022JE007346. doi: 10.1029/2022JE007346 (PMC9787587; doi:10.1029/2022JE007346)
Supplement: Supplementary file 1 — Supporting Information S1 [file JGRE-127-e2022JE007346-s001.pdf]

Supporting Information for "Impacts of Heterogeneous Chemistry on Vertical Profiles of Martian Ozone"

M. A. J. Brown[1], M. R. Patel[1,2], S. R. Lewis[1], J. A. Holmes[1], G. J. Sellers[1], P. M. Streeter[1], A. Bennaceur[1], G. Liuzzi[3,4], G. L. Villanueva[3], A. C. Vandaele[5]

[1]School of Physical Sciences, The Open University, Milton Keynes, U.K.

[2]Space Science and Technology Department, Science and Technology Facilities Council, Rutherford Appleton Laboratory, Oxfordshire, U.K.

[3]Planetary Systems Laboratory, NASA Goddard Space Flight Center, Greenbelt, MD, USA

[4]Department of Physics, American University, Washington, DC, USA

[5]Royal Belgian Institute for Space Aeronomy (BIRA-IASB), Brussels, Belgium

Contents of this file

1. Text S1 to S3
2. Figures S1 to S5
3. Tables S1 to S2

Introduction

Corresponding author: M. A. J. Brown, School of Physical Sciences, The Open University, Milton Keynes, U.K. (megan.brown@open.ac.uk)

This Supporting Information contains further information on the methodology for the cross-correlation analysis and the 1-D model chemical scheme used in the analysis. Text S1 describes the data filtering used for the observed ozone and water ice profiles and covers Tables S1 and S2. Text S2 describes the cross-correlation methodology in more detail. Text S3 explains the chemical rates used in the heterogeneous scheme and the two model comparisons undertaken to validate the 1-D model. Figures S1 to S5 are covered in Text S2.

Text S1.

1. Vertical Profiles: Units And Data Filtering

This section applies to the vertical cross-correlation in Section 2.2 of the manuscript. Water ice profiles are retrieved in ppm using pressures and temperatures from the Global Environmental Multiscale Mars (GEM-Mars) GCM (Liuzzi et al., 2020), while ozone is retrieved as number density (number of molecules / cm^3) (Patel et al., 2021). To keep the ozone and water ice data as consistent as possible and to reduce any additional errors arising from using two GCMs, both data are converted to parts per million by volume (ppmv) using the temperatures and pressures from the same GCM.

The temperatures and pressures from the Open University modelling Group Mars GCM (MGCM) dataset are used for the conversion. The MGCM is utilised for the investigation, as it has been run with data assimilation of MCS temperature and dust, and thus is optimised for this analysis. The ozone dataset is converted from the retrieved unit, molecular density, to ppmv via the ideal gas law, using data from the MGCM.

Water ice profiles are retrieved in parts per million (ppm) using temperatures and pressures from the Global Environmental Multiscale Mars (GEM-Mars) GCM (Liuzzi et

al., 2020). Therefore, in order to convert them into ppmv with MGCM temperatures and pressures, the water ice dataset is first converted into number density using the GEM-Mars GCM data, before the data is converted into ppmv with MGCM data.

Once converted into comparable units, the water ice dataset is filtered to remove data with high uncertainty (ozone filtering is described in the main analysis). The mean and median relative errors are used as they give a general summary indication of the distribution of errors. Table S1 shows the different levels of filtering applied to water ice, given the mean and median relative error, while Table S2 shows the results of the sensitivity analysis when implementing different minimum abundance requirements for the ozone and water ice profile pairs. The minimum of 6 datapoints per profile is already included in the profile pair count.

Text S2.

2. Cross-Correlation: Methodology

This section describes the cross-correlation analysis used in Section 2.2 of the manuscript. The general standardised discrete correlation is given by Chatfield (1983):

$$\mathrm{cor}(X,Y) = \frac{\mathrm{cov}(X,Y)}{\sigma_X \sigma_Y}, \tag{1}$$

where X and Y are random variables, σ is the standard deviation of those variables, and the covariance is given by:

$$\mathrm{cov}(X,Y) = \sum_{i=1}^{N} \frac{(x_i - \mu_x)(y_i - \mu_y)}{N} \tag{2}$$

where μ is the mean and N is the total number of observations. A standard correlation makes the assumption that the dependent variable (e.g. ozone) is correlated at the same point of the independent variable (e.g. water ice). For example, if x_i and y_i are two single variables from datasets X and Y, then a standard correlation only tests the relationship at point i. Two variables may have a lagged correlation, as is often the case in time series where the effects of X on Y are delayed. A variation on the standard correlation to account for this is cross-correlation and a standardised version of this is given by Chatfield (1983):

$$\mathrm{cor}_{X,Y}(z) = \frac{\sum(x - \overline{x})(y - \overline{y})}{\sqrt{\sum(x - \overline{x})^2}\sqrt{\sum(y - \overline{y})^2}} \tag{3}$$

where z is an array of lags which Y iterates through and the limits for the summations change for each lag. The results of a standardised correlation are bound between $-1 \leq \mathrm{cor}_{X,Y}(z) \leq 1$, where 1 is a perfect positive correlation, -1 is a perfect negative correlation, and 0 indicates no relationship. Correlation tests work with the assumption that any potential relationship between the variables is linear. While water ice and ozone may not fit this assumption, the analysis will provide a guidance to the patterns between the variables.

The results are then tested using a Student's T-test with a two-tailed test at a significance level $\alpha = 0.05$. The significance test assumes that the correlation follows a normal distribution and has a mean of zero and a standard variation of 1, which is a suitable assumption as the correlation values are standardised. Correlation values are converted to critical values using equation 4, which is dependent on the number of datapoints in the correlation, n_i.

$$t_i = \frac{r_i\sqrt{n-2}}{\sqrt{1-r_i^2}} \tag{4}$$

where t_i is the critical value and r_i is the correlation for the ith occultation.

For a universal comparison, the critical values are then changed into p-values; p-values less than α are deemed statistically significant. The maximum correlation for each occultation is defined by the lowest p-value, $\min(p_{i<\alpha})$, which is the most significant value.

Text S3.

3. 1-D Model: Validation Of The 1-D MPM

In order to test general variations and the quantity of species in the 1-D MPM, ozone vertical profiles are compared against outputs from both the 1-D JPL Caltech model from Viúdez-Moreiras, Saiz-Lopez, Blaszczak-Boxe, Manfredi, and Yung (2019) and the MGCM from Holmes, Lewis, Patel, and Lefèvre (2018).

For the 1-D comparison, the 1-D MPM was run with same initial temperature and water vapour profiles as given in Viúdez-Moreiras et al. (2019), under the same conditions (L_{S}= 0°, latitude ≈ 5° S) and without any heterogeneous chemistry. The model was run for 10 days to allow it to reach a steady state and allow sufficient time for the ozone abundance to stabilise.

Figure S2 shows the outputs of the 1-D MPM and the JPL Caltech model; both results show a dramatic change in ozone abundance during the sunlight hours ($\approx 0600 - 1800$ LST) > 40 km. While this is less noticeable at lower altitudes (< 30 km), there is still a diurnal variation, with an increase in ozone abundance during sunlight hours in both model runs. This is likely due to the photochemical formation of ozone, which, combined

with limited HO_x species at those altitudes, leads to an overall increase in ozone. The magnitudes of the ozone abundance between the two model outputs are similar throughout the sol with the exception of a few distinct features.

Below 20 km, the ozone abundance in the 1-D MPM is greater than the JPL Caltech ozone abundance between 0800–1600 hours. This is likely due to the lower water vapour abundances in this altitude region; while the 1-D MPM model run did have the same initial water vapour profile as the JPL Caltech run, some of the water vapour in the 1-D MPM condensed into water ice, which led to a lower concentration of HO_x than in the JPL Caltech model. Above 70 km across the whole sol, the water vapour is < 2.5 ppmv in the 1-D MPM output and even between 60–70 km the water vapour remains < 5 ppmv throughout the sol. In comparison, water vapour abundance in the JPL Caltech model varies between 60–80 km throughout the day; it is this variation which likely causes the ozone to fluctuate > 60 km while it remains, for the most part, static in the 1-D MPM above these altitudes. The decrease in ozone between 0200–0600 hours at 55 km in the 1-D MPM is likely due to the presence of H_2O_2 and HO_2, both of which decrease after 0600 hours. In the JPL Caltech model run, HO_2 abundance increases around 50–55 km in the JPL Caltech model, while the H_2O_2 abundance is not given.

Ozone abundance increases between 40–60 km from 1000 to 1800 LST in the 1-D MPM, while in the JPL Caltech model it remains constant throughout the sunlight hours. This change may be due to the increase in temperature from 1000 hours onwards, up to 50 km in the 1-D MPM, which would increase the rates of reactions and allow ozone to form at a greater rate than its destruction, which is not present in the JPL Caltech model.

Figure S3 shows the diurnal cycle of the 1-D MPM with the new heterogeneous scheme and the MGCM assimilated with temperature and dust using the old heterogeneous scheme. Both models use the offline ASIS photochemical scheme from Cariolle et al. (2017). Vertical profiles of temperature, water vapour, and water ice were taken from the MGCM and used as initial starting profiles for the 1-D model. The magnitude of the ozone abundance in both panels of Figure S3 is similar, although in the MGCM simulated ozone (right panel) has a greater abundance $> 50\,\mathrm{km}$, likely due to the horizontal transport of O-rich species (O_2 and O). Below 25 km, the ozone distribution and abundance in the 1-D MPM and MGCM are in agreement with each other, with a slightly higher concentration of ozone forming at 1200 hours near the surface in the 1-D MPM.

The largest discrepancy between the two model runs is between 30–50 km. In the MGCM simulation ,ozone abundance is between 0.1–1 ppmv, while it is < 0.1 ppmv in the 1-D MPM simulation throughout the sol. The ozone features visible in the 1-D MPM at these altitudes (30–50 km) do arguably appear in the MGCM, but at a higher altitude (> 50 km). This is likely due to the water ice distribution, as in the 1-D MPM a layer of water ice cloud forms between 25–45 km, and has very little diurnal variation. In contrast, water ice in the MGCM forms in the late evening and persists through the night before subliming into water vapour the following morning. The presence of water ice causes the adsorption of HO_x, which decreases the HO_x abundance. In the MGCM, the heterogeneous scheme converts the adsorbed HO_x species to oxygen and water vapour, which can then be recycled to influence the ozone formation or destruction. At night, the recycled oxygen can combine with molecular oxygen to form ozone, increase the ozone abundance, while during the day, the recycled water vapour can be photolysed to produce

HO_x, and thus reduce ozone abundance. The quantities of HO_x converted to H_2O and O_2 are small relative to the amount of H2O and O2 present in the column. However, they occur within the altitude ranges of water ice clouds, where they typically have a much smaller abundance. The additional formation of such species (water vapour, oxygen and, by extension, molecular oxygen) has a significant enough impact to influence the ozone abundance at these altitudes.

In the 1-D MPM, on the other hand, the HO_x species are adsorbed and converted into the adsorbed species, ice_HO_x. The adsorbed species are only released back to their original HO_x status when water ice sublimates. As water ice tends to sublimate during the day, the ice_HO_x species are also only released during the day, resulting in a decrease in ozone across all altitudes at 0600 hours at the start of the sol.

Figure S4 shows the comparison between the new heterogeneous scheme with all three reactions (OH, HO_2 and H_2O_2) (left panel) and the same heterogeneous scheme without the H_2O_2 heterogeneous reaction (right panel) at midday. While the addition of the heterogeneous H_2O_2 reaction creates an equilibrium between gas-phase and adsorbed H_2O_2 at 25–55 km (the altitude at which water ice exists), this has a minimal impact both on the hydroxyl radicals and ozone. Within this altitude range, the equilibrium favours H_2O_2 in the gas phase, and the adsorbed H_2O_2 is an order of magnitude smaller than its gaseous counterpart, leading to minimal impact on ozone and molecular oxygen ($< 1 \times 10^{-4}$ ppmv). As the equilibrium constant for H_2O_2 adsorption is exponentially dependent on temperature (Equation 4), the impact of this reaction is likely to be greater when the temperature is colder.

References

Cariolle, D., Moinat, P., Teyssèdre, H., Giraud, L., Josse, B., & Lefèvre, F. (2017). Asis v1.0: an adaptive solver for the simulation of atmospheric chemistry. *Geoscientific Model Development*, *10*(4), 1467–1485. Retrieved from https://gmd.copernicus.org/articles/10/1467/2017/ doi: 10.5194/gmd-10-1467-2017

Chatfield, C. (1983). *Statistics for technology* (Third ed.). Penguin Books Ltd.

Holmes, J. A., Lewis, S. R., Patel, M. R., & Lefèvre, F. (2018). A reanalysis of ozone on mars from assimilation of spicam observations [Journal Article]. *Icarus*, *302*, 308-318. Retrieved from http://www.sciencedirect.com/science/article/pii/S0019103517302889https://ac.els-cdn.com/S0019103517302889/1-s2.0-S0019103517302889-main.pdf?_tid=22af08de-4263-48e8-8b2a-ec0c20c3647e&acdnat=1539352983_3816efbb662a3bb2f63f199e604ffdd9 doi: https://doi.org/10.1016/j.icarus.2017.11.026

Liuzzi, G., Villanueva, G. L., Crismani, M. M., Smith, M. D., Mumma, M. J., Daerden, F., ... Patel, M. R. (2020). Strong variability of martian water ice clouds during dust storms revealed from exomars trace gas orbiter/nomad [Journal Article]. *Journal of Geophysical Research: Planets*, *125*(4), e2019JE006250. Retrieved from https://agupubs.onlinelibrary.wiley.com/doi/abs/10.1029/2019JE006250 (e2019JE006250 2019JE006250) doi: 10.1029/2019JE006250

Patel, M. R., Sellers, G., Mason, J. P., Holmes, J. A., Brown, M. A. J., Lewis, S. R., ... Lopez-Moreno, J.-J. (2021). Exomars tgo/nomad-uvis vertical profiles of ozone: 1. seasonal variation and comparison to water. *Journal of Geophysical Research: Planets*, *126*(11), e2021JE006837. Retrieved from https://agupubs

.onlinelibrary.wiley.com/doi/abs/10.1029/2021JE006837 (e2021JE006837 2021JE006837) doi: https://doi.org/10.1029/2021JE006837

Viúdez-Moreiras, D., Saiz-Lopez, A., Blaszczak-Boxe, C., Manfredi, J. R., & Yung, Y. (2019). Diurnal variation in mars equatorial odd oxygen species: Chemical production and loss mechanisms. *Icarus*, 113458.

Table S1. The errors used are taken from the retrieved data from Liuzzi et al. (2020). The median and mean error given by using varying thresholds for the water ice (H_2O) data, as well as the number of datapoints used after applying this threshold. The last column displays this as a percentage of the total datapoints. Threshold used in the manuscript is highlighted in red.

Threshold	Median error	Mean error	No. of datapoints	% of datapoints used
No filter	0.40	4.81	289855	100
$\frac{H_2O}{H_2O_{error}} > 1$	0.32	4.32	178920	61.7
$\frac{H_2O}{H_2O_{error}} > 4$	0.22	3.23	121045	41.8
$\frac{H_2O+1}{H_2O_{error}} > 4$	0.16	2.16	188819	65.1
$\frac{H_2O+1}{H_2O_{error}} > 3$	0.18	2.43	204623	70.6
$\frac{H_2O+1}{H_2O_{error}} > 5$	0.14	1.90	175122	60.4

Table S2. Number of available vertical profile pairs when placing restrictions on the minimum threshold. At least one datapoint within a profile must be higher than the restriction value for the profile to be included in the analysis. The minimum number of datapoints – 6 per profile – is already accounted for. Threshold used in the manuscript is highlighted in red.

Ozone Limit (ppmv)	Water Ice Limit (ppmv)	No. Vertical Profiles	Percent of Total Profiles (%)
None	None	1085	100
0.01	None	1082	99.7
0.02	None	1075	99.1
0.03	None	1029	94.8
0.04	None	953	87.8
0.05	None	881	81.2
None	0.1	1057	97.4
None	0.2	1040	95.9
None	0.5	964	88.8
None	1	854	78.8
None	1.5	705	65.0
None	2	604	55.7
0.03	1	813	74.9

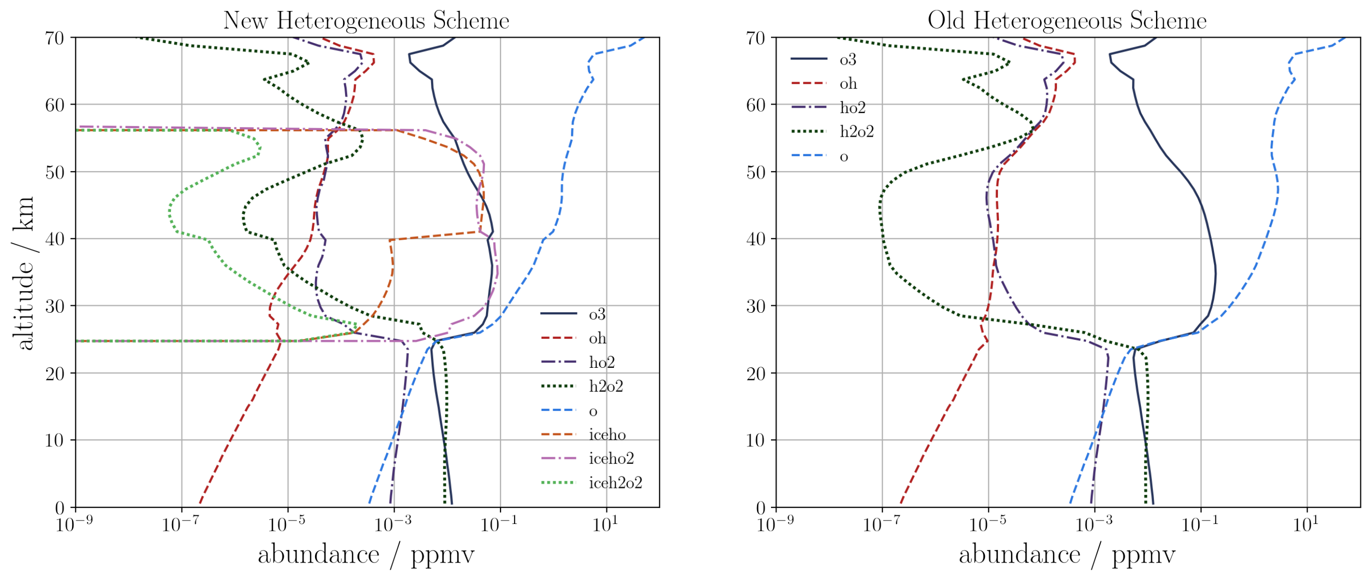

Figure S1. Abundance of the main chemical species affecting ozone taken at 1200 hours, latitude 0° S, L_S= 5° with the 1-D MPM (left) run with the new heterogeneous chemistry scheme and (right) the old heterogeneous chemistry.

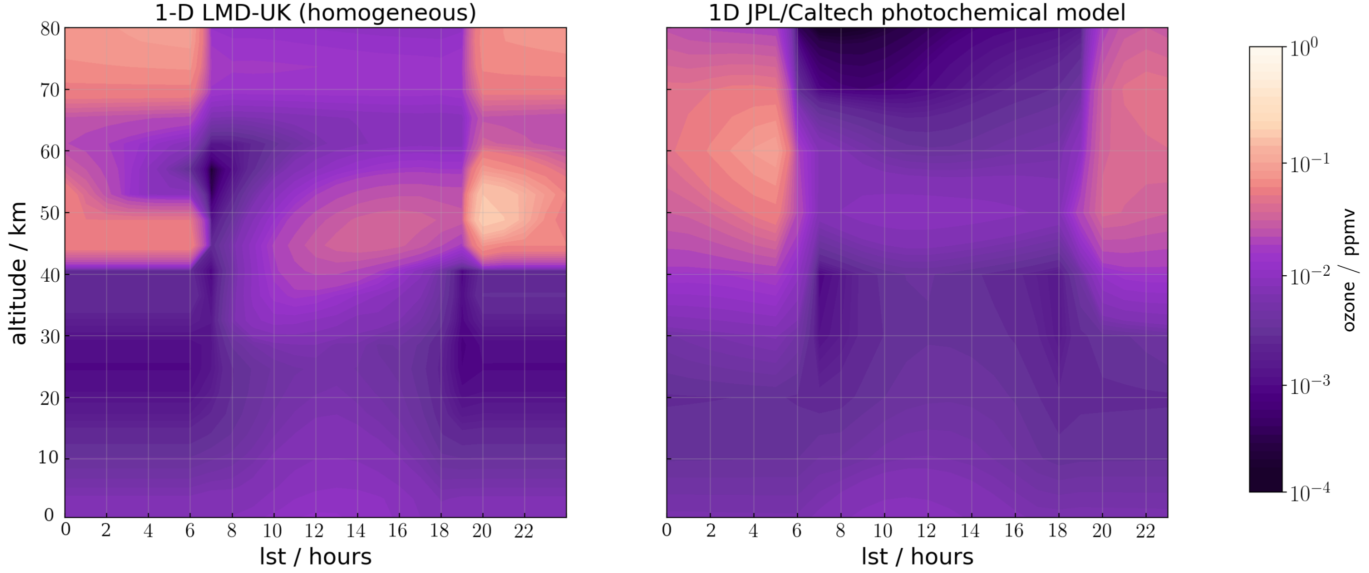

Figure S2. Ozone abundance over a sol at 5° S, L_S= 0° with; (left) the 1-D MPM and; (right) the JPL Caltech model (Viúdez-Moreiras et al., 2019).

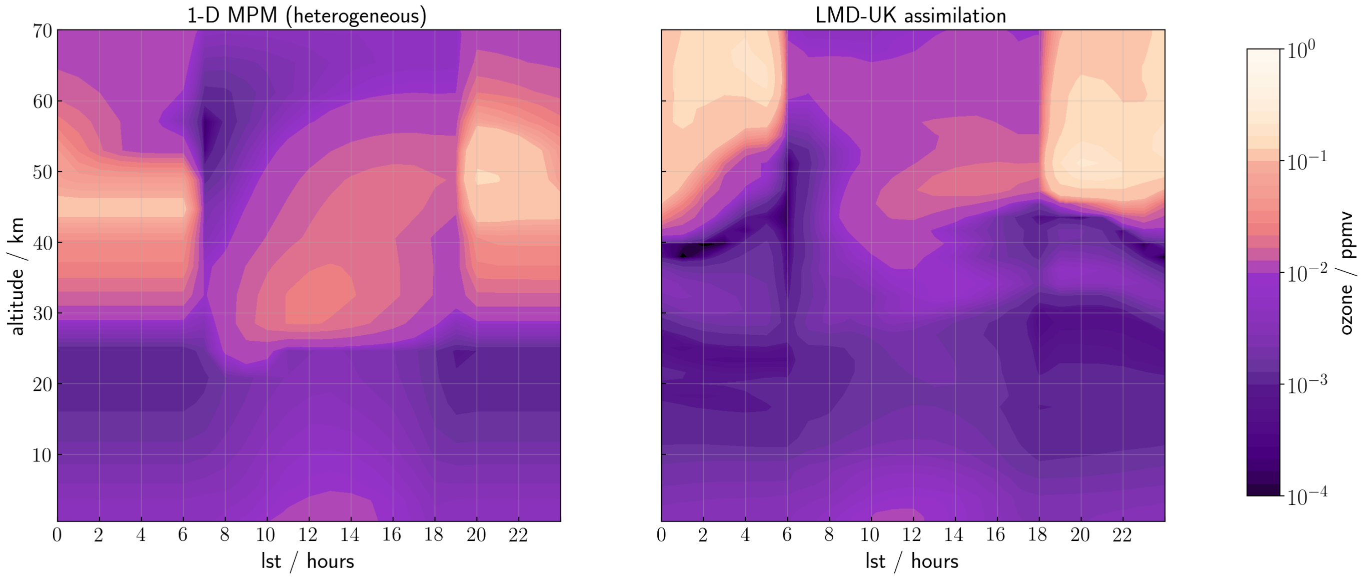

Figure S3. Ozone abundance over a sol at 0° S, L_S= 15° with (left) the 1-D MPM (run with heterogeneous chemistry) and (right) the MGCM with assimilated temperature and dust using the offline ASIS scheme taken from Holmes et al. (2018).

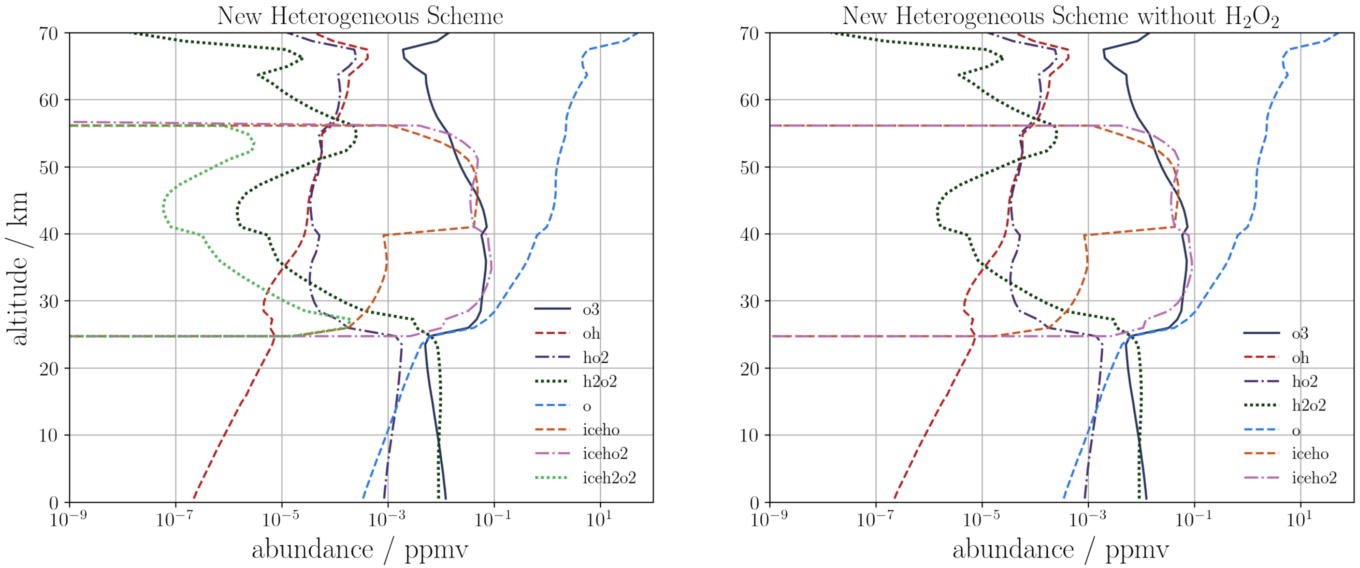

Figure S4. Ozone abundance over a sol at 0° S, L_S= 15° with the 1-D MPM using; (left) the new, full heterogeneous scheme; (right) the new heterogeneous scheme without the H_2O_2 adsorption reaction.
